# Kidney Trouble the Cause of Toothache

**Published:** 1883-10

**Authors:** 


### KIDNEY TROUBLE THE CAUSE OF TOOTHACHE.

This kind of toothache often accompanies gravel in kidney or bladder, and is of the most acute agonizing form. None of the usual remedies can be employed, not even the extraction of the tooth will obliterate the pain, which generally disappears as suddenly as it came. Dr. Rodemacher recommends the use of cochineal against the kidney gravel, and as this disappears, the toothache leaves without any medicine.—*Ibid.*

